# Crossover effect of spouse weekly working hours on estimated 10-years risk of cardiovascular disease

**DOI:** 10.1371/journal.pone.0182010

**Published:** 2017-08-03

**Authors:** Mo-Yeol Kang, Yun-Chul Hong

**Affiliations:** 1 Department of Preventive Medicine, Seoul National University College of Medicine, Seoul, Republic of Korea; 2 Institute of Environmental Medicine, Seoul National University Medical Research Center, Seoul, Republic of Korea; Universidad Miguel Hernandez de Elche, SPAIN

## Abstract

**Objectives:**

To investigate the association between spouse weekly working hours (SWWH) and the estimated 10-years risk of cardiovascular disease (CVD).

**Methods:**

This cross-sectional study was based on the data obtained from the Korean National Health and Nutrition Examination Survey 2007–2012. Data of 16,917 participants (8,330 husbands, 8,587 wives) were used for this analysis. The participants’ clinical data were collected to estimate the 10-years risk of CVD, as well as weekly working hours. Multiple logistic regression was conducted to investigate the association between SWWH and the estimated 10-years risk of CVD. We also performed a stratified analysis according to each participant’s and their spouse’s employment status.

**Results:**

Compared to those whose spouses worked 30 hours per week, estimated 10-years risk of CVD was significantly higher as SWWH increase among those whose spouses worked >30 hours per week. After adjusting for covariates, the odds ratio for high CVD risk was found to increase as SWWH increased, up to 2.52 among husbands and 2.43 among wives. We also found that the association between SWWH and the estimated 10-years risk of CVD varied according to the employment status. Analysis of each component included in the CVD appraisal model showed that SWWH had close relationship with diabetes in men, and smoking habits in women.

**Conclusions:**

Spouse’s long working hours are associated with individual’s risk of CVD in future, especially among husbands.

## Introduction

There is sufficient evidence to prove the association between the incidences of mental and physical health problems and individual’s working hours [1, 2]. However, few studies have concentrated on the possible effect of working hours on the spouse, the person with whom the worker most frequently interacts. A large body of studies in the area of occupational psychology has shown that poor working conditions can worsen employees’ family life [3].Moreover, it has been reported that workplace stress and strain affect not only the person’s own health but transmitted to their partners as well, both in terms of mental and physical health [3].

The association between cardiovascular disease (CVD) and long working hours has been investigated in several studies [1, 2]. Golden and Wiens-Tuers suggested that a work-family life imbalance may be one of the key factors in associations between working overtime and adverse cardiac health [4]. They also suggested that the adverse effects of long working hours, such as fatigue, work stress, and work-family interference, were not offset by higher income. Individuals who work overtime may be unsatisfied with their work-life imbalance, which can ultimately affect every area of their lives, including domestic relationships. In a prospective cohort study done on full-time municipal employees, higher rates of sickness-related absences, psychological distress, and poor health were observed among those who experience severe work-family conflict than those who did not have such experiences [5].

Further research, pertaining to the dyadic nature of stress crossover to the spouse, is required, because they might cause adverse reciprocal effects between the couples, leading to a spiral loss of resource and impaired functionality[6]. In recent times, there has been an increase in the number of studies that have focused on the crossover effect between couples, concerning depression [7], burnout [8], physical health [9], negative moods [10], and daily happiness [11]. However, most of these studies have assessed relatively small and specific occupational groups or have used data collected from Western populations. Moreover, as far as we know, there is no direct evidence supporting the theory that working overtime can affect a spouse’s future risk of CVD.

In our study, we investigated the crossover effect of long working hours on Korean couples, particularly in terms of cardiovascular health. The primary purpose of this study was to investigate the relationship between the spouse working hours and estimated 10-years risk of CVD, using representative data from a national population-based survey in Korea. In addition, identifying the characteristics to modify this relationship would be helpful for the development of strategies to prevent CVD of workers. Therefore, we also examined how this relationship is modified by gender and employment status.

## Subjects and methods

### Study design and participants

In this study, we utilized data from the Korean National Health and Nutrition Examination Survey (KNHANES). The KNHANES has been conducted by the Korea Center for Disease Control and Prevention (KCDC) since 1998. The KCDC conducted the 4^th^ KNHANES from 2007 to 2009 and the 5^th^ KNHANES from 2010 to 2012. These yearly data are available in the KNHANES website (https://knhanes.cdc.go.kr/knhanes/eng/index.do). Multistage probability sampling was used, stratified by geographic location, sex, and age. The original study was approved by the Institutional Review Board (IRB) of the KCDC (IRB: 2007-02-CON-04-P; 2008-04EXP-01-C; 2009-01CON-03-2C; 2010-02CON-21-C; 2011-02CON-06-C; 2012-01EXP-01-2C).

At the time of the KNHANES 2007–2012, citizens were informed that they had been randomly selected as a household to voluntarily participate in the nationally representative survey conducted by the Korean Ministry of Health and Welfare. All participants of the KNHANES provided written informed consent. The KNHANES collects information on the participants’ socioeconomic status, and the participants undergo anthropometric measurements, a health interview, a physical examination, and a nutrition survey, through the face-to-face interview.

**Fig 1:**
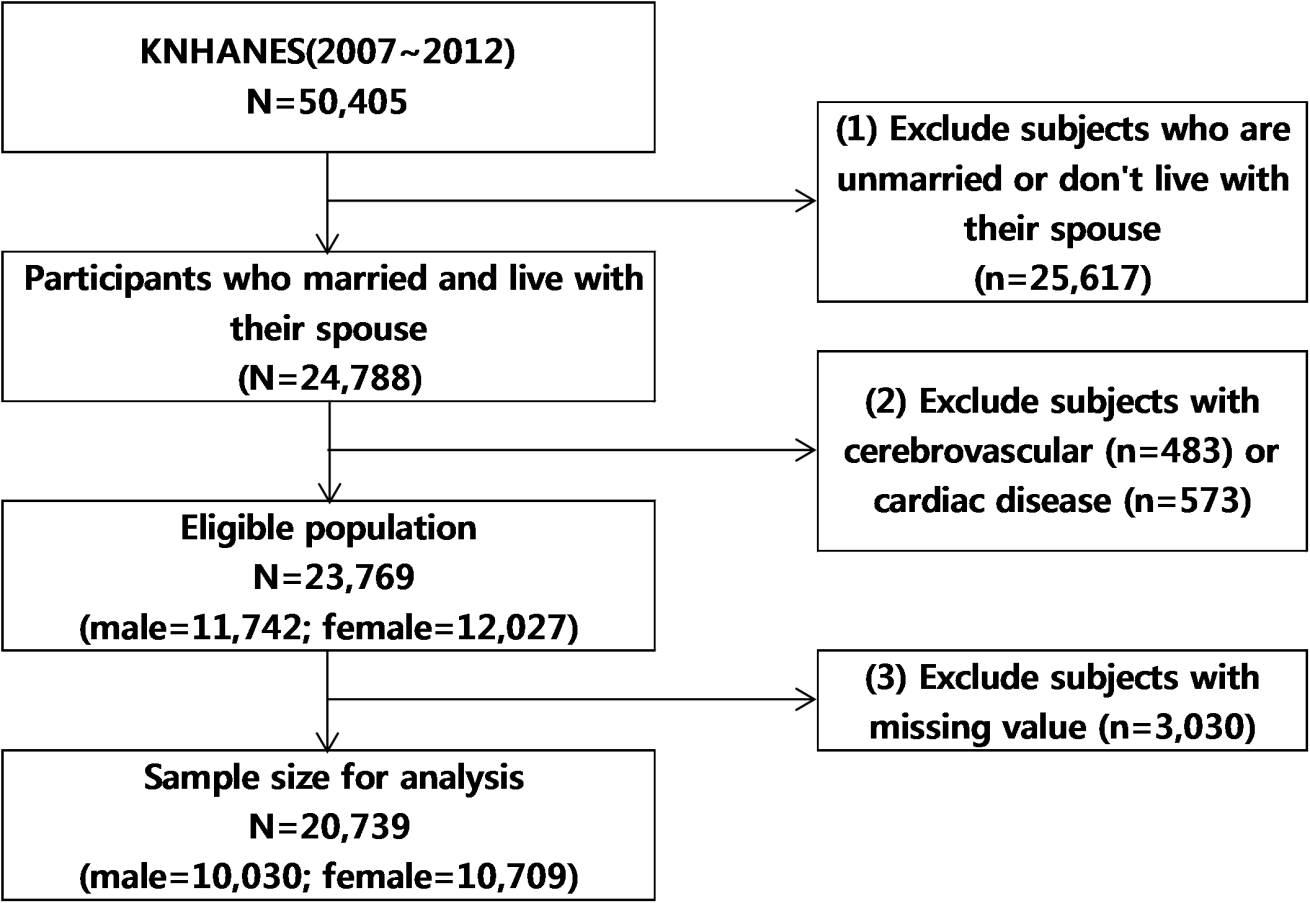
Schematic diagram depicting study population.

In total, 50,405 individuals (24,871 and 25,534 individuals from the 4^th^ and the 5^th^ KNHANES, respectively) participated in the surveys. Because sampling units of KNHANES were households, couples were matched with their household identification numbers. For our analysis, we included participants who were married and lived with their spouse during the KNHANES (n = 24,788). After excluding participants with cerebrovascular disease (n = 483) or cardiac disease (n = 573), 23,769 participants (11,742 men, 12,027 women) were eligible for participation. The final sample for analysis included 20,739 participants (10,030 husbands, 10,709 wives), after further excluding participants with missing values (n = 3,030) (Fig 1).

### Measurements

The KNHANES included questions pertaining to a wide array of characteristics. Trained staff members reviewed the completed questionnaires and entered them into a database. We used the variables for age, sex, occupation, medical history, and smoking habit. Occupations were classified into 7 groups: ‘Managers and professionals’, ‘Office workers’, ‘Service and sales workers’, ‘Agriculture, forestry and fishery workers’, ‘Craft, device machine operators and assembly workers’, ‘Manual workers’, and ‘Unemployed’. Weekly working hours were measured as the actual number of hours the respondent worked per week across all paid jobs.

We defined the non-smoker was those who had either never smoked or were ex-smokers. Participants’ blood pressure was measured in the right arm at heart level using a standard mercury sphygmomanometer (Baumanometer, USA) while they were seated and after they had rested for at least 5 min. The average of two measured value each for systolic blood pressure (SBP) and diastolic blood pressure (DBP), which were measured at an interval of 5 min, was used for analysis. After fasting 12-hr overnight, blood samples were obtained from an antecubital vein. Total cholesterol and high-density lipoprotein (HDL) cholesterol were measured using an autoanalyzer (ADVIA 16501, Bayer, Tarrytown, NY, USA).

The individual risk for CVD was determined using a health risk appraisal model for coronary heart disease, which was based on data collected nationwide from the Korean Heart Study, which included 430,920 individuals (266,782 men, 164,138 women) combined with National Health Insurance System [12]. This model was developed based on the multivariate Cox proportional hazard model using a retrospective cohort data in the same manner as in the Framingham equation model [13]. This model was validated using another sample of the representative population who received health insurance from the National Health Insurance System [14]. The first step in estimating the future risk of CVD was to calculate the score for each risk factor, after which an individual’s 10-year risk was computed using cardiovascular risk functions specific to Korean men and women. Assessment of global risk for CVD based on the summation of categorical values of major risk factors: age, sex, total cholesterol, high-density lipoprotein (HDL) cholesterol, blood pressure, diabetes, and smoking. Next, participants were stratified into two groups: high-risk group and low-risk group. We defined the predicted risk of fatal and non-fatal CVD events of 90^th^ percentile or greater by gender as the threshold for high risk among study population.

### Statistical analysis

The general characteristics of the study population (10,030 men, 10,709 women) were described using mean (± standard deviation) or frequencies and percentages. The means of estimated 10-years risk of CVD was calculated by spouse weekly working hours (SWWH) and both partners employment status. After excluding subjects who lacks information about SWWH (spouse’s unemployment or missing value), 12,991 subjects (4,820 husbands, 8,171 wives) stratified into high and low CVD risk were categorized by SWWH. Multiple logistic regression analyses were used to evaluate relationships between high CVD risk and SWWH categories according to sex, adjusting for household income level, employment status, own weekly working hours, and spouse occupation categories. Given that the results could be affected by gender and both partners employment status, we performed stratified analysis according to each participant and the spouse employment status, using entire analytic population (10,030 men, 10,709 women) except for subjects with missing value.

The numbers of subjects were different between eligible population and analytic population across the tables. This was due to dyadic property of our study. For example, there were cases in which analysis of wife was possible, but the analysis of the husbands was not possible due to a lack of information on wife’s working hour while we had the information for husbands. Moreover, 1,496 subjects (701 husbands, 795 wives) were not used in main analysis because of missing value about estimated 10-years risk of CVD, despite the presence of information on SWWH. Since 10-years risk of CVD was estimated by combining five risk factors, participants should have been excluded from the final analytic models even when only one value was missing. So, we conducted additional analyses including 1,478 subjects, in which the missing values were treated using multiple imputation technique [15]. We also analyzed each component included in CVD appraisal model by SWWH categories.

Two-tailed p-values <0.05 were considered statistically significant. Statistical analyses were performed using SURVEYREG and SURVEYLOGISTIC in SAS (ver. 9.3, SAS Institute, Cary, NC, USA), a software package that account for the complex sample design. Survey sample weights were used in all analyses to produce estimates that were representative of the Korean population. Figures were drawn by using a generalized additive model (GAM) of R version 3.2.4.

## Results

Mean ages of the male and female participants were 51.8 (± 13.5) and 48.7 (± 12.9) years, respectively. The levels of total cholesterol were similar between men and women, but HDL levels were slightly lower in male participants than in female participants. The other risk factors for CVD such as high blood pressure, diabetes mellitus, and current smoking, were higher in the male participants than for the female participants. The proportion of each sex that was unemployed was 17.1% in men and 50.0% in women. The largest percentage of participants worked 40–49 hours per week for men, and <30 hours for women. The descriptive characteristics of the study population are summarized in Table 1.

**Table 1:** Demographic and clinical characteristics of study populations.

	Male		Female	
	Mean	SD	Mean	SD
Age	51.8	13.5	48.7	12.9
Total cholesterol (mg/dl)	190.4	34.9	190.7	36.6
HDL cholesterol (mg/dl)	48.0	11.9	53.7	12.8
SBP (mmHg)	121.6	15.9	115.8	17.4
DBP (mmHg)	79.5	10.7	74.2	10.0
	**n**	**%**	**n**	**%**
Diabetes				
Yes	1146	11.9	712	7.2
No	8496	88.1	9110	92.8
Smoking				
Non-smoker	1643	16.7	9575	92.2
Ex-smoker	4259	42.2	465	4.5
Current smoker	4157	41.2	348	3.4
Occupation				
Managers and professionals	1828	18.1	897	8.6
Office workers	1222	12.1	614	5.9
Service and sales workers	1195	11.8	1470	14.1
Agriculture, Forestry and Fisheries Workers	1189	11.8	952	9.2
Craft, device machine operators and assembly workers	2189	21.6	316	3.0
Manual workers	758	7.5	949	9.1
Unemployed	1734	17.1	5195	50.0
Working Hours				
<30	818	9.7	1191	23.1
30–39	733	8.7	812	15.7
40	1445	17.2	724	14.0
41–49	1635	19.4	862	16.7
50–59	1636	19.5	623	12.0
60–69	1086	12.9	409	7.8
70–79	668	7.9	332	6.4
≥80	389	4.6	210	4.1
**Total**	10,030		10,709	

**Fig 2:**
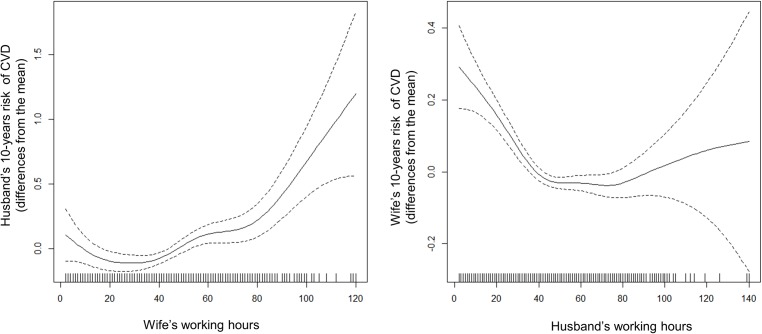
Generalized additive model of spouse’s working hours and estimated 10-years risk of CVD. 10-years risk of CVD predicted was estimated by Jee’s health risk appraisal model. The values of Y-axis indicate differences from the means. Household income level, employment status, own weekly working hours, and spouse occupational category are adjusted.

After excluding subjects without information on SWWH, estimated 10-years risk of CVD for 4,820 husbands and 8,171 wives are presented according to SWWH in Table 2. The lowest risk of CVD was observed in the 40 SWWH group for both men and women. After adjusting for household income level, employment status, own weekly working hours, and spouse occupational category, it was observed that the risk for CVD was lowest among those whose spouses worked 40 hours in both gender (Fig 2). Generally, the probability of being high risk group for CVD in men increased as the SWWH increased. Moreover, the results showed a dose-response relationship, suggesting that the longer spouse worked, the more likely individual become high risk group for CVD (≥80 hours, men, OR = 2.52; women, OR = 2.43). Those whose spouse worked < 40 hours showed higher values than those whose spouse worked 40 hours. In summary, Table 2 displays that the overall risk of CVD was higher among men than women, and the association between SWWH and estimated 10-years risk of CVD was more remarkable in men (men, p for trend = 0.007; women, p for trend = 0.089).

**Table 2:** 10-years risk of CVD estimated by Jee’s health risk appraisal model according to spouses’ working hour categories.

	Spouse's Working Hours	N	Estimated 10-years risk		≥ 90 percentile of estimated risk of cardiovascular disease			
			Mean	SD	N	%	OR*	95% CI
Husband’s 10-year Risk of CVD According Wife’s Working Hour Categories	<30	1092	1.73	1.71	96	8.79	1.13	0.66–1.92
	30–39	746	1.66	1.58	58	7.77	1.39	0.80–2.43
	40	682	1.24	1.20	28	4.11	1	Reference
	41–49	811	1.61	1.58	65	8.01	1.73	1.00–2.97
	50–59	593	1.74	1.44	46	7.76	1.76	1.00–3.12
	60–69	390	2.05	1.93	45	11.54	1.93	1.05–3.55
	70–79	311	1.81	1.29	23	7.40	1.38	0.69–2.78
	≥80	195	2.13	1.69	22	11.28	2.52	1.20–5.32
	Total	4820	1.74	1.75	383	7.95	*p for trend = 0*.*007*	
Wife’s 10-year Risk of CVD According husband’s Working Hour Categories	<30	770	0.87	1.01	155	20.13	5.20	2.59–10.43
	30–39	713	0.51	0.74	67	9.40	3.91	1.87–8.14
	40	1412	0.24	0.36	26	1.84	1	Reference
	41–49	1590	0.30	0.57	67	4.21	2.02	0.97–4.19
	50–59	1601	0.31	0.59	60	3.75	2.06	0.99–4.26
	60–69	1064	0.32	0.48	51	4.79	3.03	1.47–6.25
	70–79	651	0.42	0.88	34	5.22	2.04	0.92–4.50
	≥80	370	0.41	0.63	25	6.76	2.43	1.05–5.66
	Total	8171	0.52	0.83	485	5.94	*p for trend (≥40 hrs) = 0*.*089*	

*adjusted for household income level, employment status, own working hours and spouse occupation categories

We also found that the association between SWWH and the estimated 10-years risk of CVD varied according to the couple’s employment status (Tables 3 and 4). In general, unemployed men had more than a 2-fold higher risk of CVD within 10 years than employed men (estimated 10-years risk for CVD, employed men = 1.43; unemployed men = 3.27) (Table 3). When their wives were unemployed, employed husbands’ 10-year risk of CVD was 1.31% and unemployed husbands’ risk was 3.46%. A dose-response relationship between wives’ working hours and estimated 10-years risk of CVD was observed only among employed husbands (p for trend<0.001), but not among unemployed husbands. In contrast, association between SWWH and the CVD risk of wives did not significantly differ by their spouses’ employment status (Table 4). An interesting finding to emerge from the data comparison was that the highest risk was observed when both wife and husband were unemployed (male, 10-year risk = 3.46%; female, 10-year risk = 1.35%).

**Table 3:** Husband's 10-years risk of CVD estimated by Jee’s health risk appraisal model according to their own employment status and wife’s working hour categories.

Husband's Employment status	Wife's Working Hours	N	Estimated 10-years risk		≥ 90 percentile of estimated risk of cardiovascular disease			
			Mean	SD	N	%	OR	95% CI
Employed^a^	Unemployed ^c^	3711	1.31	1.39	197	5.31	0.61	0.35–1.04
	<30	946	1.49	1.43	55	5.81	1.15	0.67–1.95
	30–39	667	1.51	1.41	41	6.15	1.41	0.81–2.45
	40	629	1.14	1.09	21	3.34	1	Reference
	41–49	728	1.48	1.38	45	6.18	1.72	1.00–2.96
	50–59	525	1.65	1.39	36	6.86	1.75	0.99–3.09
	60–69	343	1.85	1.73	28	8.16	1.90	0.03–3.48
	70–79	283	1.71	1.20	16	5.65	1.35	0.67–2.70
	≥80	166	1.97	1.57	13	7.83	2.42	1.15–5.07
	Total	8338	1.43	1.41	452	5.65		
Unemployed^b^	Unemployed ^d^	1043	3.46	2.44	338	32.41	5.20	2.59–10.43
	<30	144	3.32	2.41	41	28.47	3.91	1.87–8.14
	30–39	76	2.99	2.22	17	22.37	2.02	0.97–4.19
	40	53	2.46	1.67	7	13.21	1	Reference
	41–49	83	2.82	2.42	20	24.10	2.02	0.97–4.19
	50–59	67	2.46	1.61	10	14.93	2.06	0.99–4.26
	60–69	47	3.51	2.61	17	36.17	3.03	1.47–6.25
	70–79	28	2.89	1.65	7	25.00	2.04	0.92–4.50
	≥80	29	3.00	1.66	9	31.03	2.43	1.05–5.66
	Total	1692	3.27	2.24	466	29.68		

^a^adjusted for spouse age, income level, employment status, spouse occupation categories, and own weekly working hours

^b^adjusted for spouse age, income level, employment status, spouse occupation categories, but not own weekly working hours

^c^adjusted for spouse age, income level, employment status, own weekly working hours but not spouse occupation categories

^d^adjusted for spouse age, income level, employment status, but not own weekly working hours or spouse occupation categories

**Table 4:** Wife's 10-years risk of CVD estimated by Jee’s health risk appraisal model according to their own employment status and husband's working hour categories.

Wife's Employment status	Husband's Working Hours	N	Estimated 10-year risk		≥ 90 percentile of estimated risk of cardiovascular disease			
			Mean	SD	N	%	OR	95% CI
Employed^a^	Unemployed ^c^	548	0.88	1.00	107	19.53	3.47	1.63–7.37
	<30	427	0.79	0.88	75	17.56	5.22	2.61–10.46
	30–39	395	0.50	0.63	37	9.37	3.90	1.87–8.13
	40	678	0.22	0.32	10	1.47	1	Reference
	41–49	831	0.32	0.61	35	4.21	2.04	0.98–4.22
	50–59	828	0.36	0.59	36	4.35	2.13	1.03–4.40
	60–69	576	0.37	0.52	39	6.77	3.17	1.54–6.54
	70–79	396	0.40	0.50	21	5.30	2.20	1.00–4.84
	≥80	248	0.43	0.65	16	6.45	2.67	1.15–6.21
	Total	5347	0.45	0.68	376	7.63		
Unemployed^b^	Unemployed ^d^	1063	1.35	1.21	361	33.96	7.01	3.68–13.36
	<30	342	0.98	1.15	80	23.39	5.42	2.96–9.92
	30–39	318	0.53	0.85	30	9.43	2.47	1.26–4.84
	40	731	0.25	0.39	16	2.19	1	Reference
	41–49	756	0.29	0.52	32	4.23	1.49	0.78–2.85
	50–59	767	0.25	0.59	22	2.87	1.20	0.60–2.38
	60–69	486	0.25	0.40	12	2.47	0.87	0.39–1.91
	70–79	253	0.44	1.26	13	5.14	1.61	0.73–3.56
	≥80	121	0.36	0.58	9	7.44	2.14	0.85–5.41
	Total	5362	0.58	0.95	575	11.89		

^a^adjusted for spouse age, income level, employment status, spouse occupation categories, and own weekly working hours

^b^adjusted for spouse age, income level, employment status, spouse occupation categories, but not own weekly working hours

^c^adjusted for spouse age, income level, employment status, own weekly working hours but not spouse occupation categories

^d^adjusted for spouse age, income level, employment status, but not own weekly working hours or spouse occupation categories

## Discussion

The first main finding of the present study was that there was a close relationship between SWWH and estimated 10-years risk of CVD. This was particularly marked in the male participants of this study. These findings suggest that when an individual works overtime, it negatively affects not only his/her cardiovascular system, but also that of his/her spouse. Furthermore, the results of our study showed that couple’s employment status might modify the effects of SWWH on the risk of CVD.

Previous studies have identified two different ways in which stress is transferred: spillover and crossover. While spillover effect is a transmission of strain from one domain of life to another within a person [16], the crossover effect involves the transmission of stress and strain from an individual to his/her spouse [17]. Work-life imbalance can crossover from one spouse to another. Therefore, stress that originates in the workplace may spill over to the family domain, which, in turn, transmit to spouse by crossover effect [16]. In a study among Japanese dual-earner couples, it was reported that dual experiences of work-to-family conflict have a detrimental effect on the health of workers and the relationship between partners [18]. Matthews et al. also examined crossover effects in dual-earner couples. The results indicated that worker’s work-to-relationship conflict and the perception of partner's work-to-family conflict were associated with the worker’s as well as partner’s outcomes, such as tension of relationship, health symptoms, and the satisfaction in the relationship [19].

Perceived stress transmitted from the spouse can activate an individual’s hypothalamic pituitary adrenal (HPA) axis. Activation of inadequate or excessive adrenocortical and autonomic function can cause deleterious effect on health. Repetti et al. reported that husbands had elevated levels of cortisol, which is a stress hormone, at home after socially stressful days at work, consistent with a physiological spillover effect [20]. In a 3-day study of couples, it was found that husbands' and wives' fluctuations in negative mood and cortisol levels were linked [21].

Our finding that unemployment of both spouses increased the risk of CVD might be explained by common stressors (e.g., financial pressures or life events) which the couples share [17, 22] (Tables 3 and 4). This finding supports the idea that couples’ financial hardship can affect the perceived health status in both spouses [9]. In addition, Westman and Vinokur (1998) suggested that common life events for husbands and wives affected the crossover process by increasing each spouse’s depression [23]. In this context, unemployment does not merely mean being zero working hours, it might imply different aspects, such as financial problems, poor self-efficacy, and loss of social network. Therefore, further research into crossover effect on spouse’s job loss considering such aspects is thus warranted.

In our study, the male participants worked for an average of 48.7 (± 17.3) hours, while female participants worked for 42.5 (± 19.7) hours. We found that 45.4% (n = 3,420) of the men and 31.6% (n = 1,447) of the women worked for ≥50 hours per week. Remarkably, the association between SWWH and the risk of CVD was more prominent in husbands, especially in case of increasing of SWWH. The figures drawn by using GAM shows that the slope of husband’s CVD risk was steeper than that of wife’s, as SWWH increased (Fig 2). These findings are consistent with those of previous studies [24, 25]. Most research regarding the crossover of stress between spouses takes gender differences into account. Spitze (1988) offered interesting insights into the possible consequences of women’s engagement in work on other family members physical health [24]. Career women might have more money to pay for care, but less time available to provide physical care. Indeed, wife’s long working hours might lead to poorer household circumstances for other family members (e.g., irregular daily meals, poor nutritional balance, and less time for family-oriented leisure activities at home); therefore, this can result in adverse consequences on husbands’ cardiac health. In addition, several findings regarding the distribution of household labor generally suggest that when wives work, it leads to a greater participation of their husbands in the household, increasing relative domestic burden of the husbands [25].

The traditional expectations for gender roles play an important role in understanding the work-family conflicts across diverse cultures. The traditional gender role stereotypes assume that husbands as breadwinners are primarily responsible for family income and therefore have a greater value on the work domain, while wives are expected to take on more responsibility for family demands. East Asian culture, especially, encourages husbands to devote more time to the job to maximize the economic benefits for the family. According to this family-based work ethic, husbands’ overtime work and temporary sacrifice of the family life would be perceived as ‘normal’ and acceptable by the family members; however, they tend to have a lower threshold of acceptance for wives’ long working hours [26]. Our finding, that employed husbands whose wives were unemployed or worked less hours have a decreased risk of CVD, is consistent with the traditional gender role expectations (Table 3).

There are several limitations to this study that need to be acknowledged. First, our findings are limited by the use of a cross sectional design. Caution is warranted before definite conclusions for causal relationship between SWWH and the risk of CVD are arrived at. A reverse causation could not be excluded. For instance, individuals whose spouse had pre-existing health problems could have reduced their working hours to take care of them. To minimize these effects, we excluded participants with cerebrovascular or cardiovascular disease in our analysis. Second, lifestyle or family roles may have affected the stress response and eventually the disease development; however, we could not take this fact into consideration due to la lack of sufficient information available in our dataset. Finally, since we estimated 10-years risk of CVD by combining five risk factors, participants should have been excluded from the final analytic models even when only one value was missing. However, there were no significant differences in patterns of the relationships when we conducted additional analyses, in which the missing values were treated using multiple imputation technique (S1 Table).

Nevertheless, the present study had the following important strengths. First, it assessed a representative sample of the general population in Korea. Second, to our best knowledge, our study is the first to investigate the crossover effect of spouse long working hours on an individual’s risk of CVD, which can be used as scientific evidence for stress interventions targeting couple dyads. Third, the stratified analysis by spouse employment status and household income level provided a better opportunity to understand the mechanism and identify populations at risk, in order to develop developing practical prevention strategies.

When we analyzed each component of CVD appraisal model, we observed that SWWH had a close relationship with the risk factors of CVD such as blood pressure, cholesterol levels, diabetes, and smoking habits (S2 Table). In particular, the difference in the prevalence of diabetes in male subjects and that of smoking in female subjects by SWWH categories is outstanding compared to the other risk factors of CVD. We hope that these findings also contribute to the proper management for worker’s family as well as themselves.

In conclusion, it is suggested that the spouse’s working hours have significant association with the individual’s estimated 10-years risk of CVD, especially among husbands. These findings indicate that someone’s overtime work is adversely associated not only with their risk of CVD but also that of their spouse. Furthermore, couple’s employment status may modify this crossover effect of SWWH on the 10-years risk of CVD. Our study provides important practical implication that can be helpful for employers and employees seeking work-family balance. In terms of prevention, our results suggest that managerial supports to reduce overtime work may serve a dual purpose of improving both employee and their spouse health. In addition, providing counselling services and a supportive social environment may help employees retain work–life balance and manage health of themselves and their partners. An intervention study, therefore, is required to establish how these prevention measures would work in practice.

## Supporting information

S1 Table 10-year risk of CHD estimated by Jee's appraisal model according to spouse working hour categories including subjects with missing values, which are treated by multiple imputation. (DOCX)

S2 Table Cardiovascular risk profiles used in Jee's appraisal model according to spouse working hour categories. (DOCX)

## References

[1] Caruso CC, Hitchcock EM, Dick RB, Russo JM, Schmit JM. Overtime and extended work shifts: recent findings on illnesses, injuries, and health behaviors: US Department of Health and Human Services, Centers for Disease Control and Prevention, National Institute for Occupational Safety and Health; 2004.

[2] Kivimäki M, Jokela M, Nyberg ST, Singh-Manoux A, Fransson EI, Alfredsson L, et al Long working hours and risk of coronary heart disease and stroke: a systematic review and meta-analysis of published and unpublished data for 603 838 individuals. The Lancet. 2015;386(10005):1739–46. 10.1016/S0140-6736(15)60295-1 26298822

[3] Bianchi SM, Milkie MA. Work and family research in the first decade of the 21st century. Journal of Marriage and Family. 2010;72(3):705–25.

[4] Golden L, Wiens-Tuers B. To your happiness? Extra hours of labor supply and worker well-being. The Journal of Socio-Economics. 2006;35(2):382–97.

[5] Väänänen A, Buunk BP, Kivimäki M, Pentti J, Vahtera J. When it is better to give than to receive: long-term health effects of perceived reciprocity in support exchange. Journal of personality and social psychology. 2005;89(2):176 doi: 10.1037/0022-3514.89.2.176 16162052 10.1037/0022-3514.89.2.176

[6] Westman M, Brough P, Kalliath T. Expert commentary on work–life balance and crossover of emotions and experiences: Theoretical and practice advancements. Journal of Organizational Behavior. 2009;30(5):587–95.

[7] Hammer LB, Cullen JC, Neal MB, Sinclair RR, Shafiro MV. The longitudinal effects of work-family conflict and positive spillover on depressive symptoms among dual-earner couples. Journal of Occupational Health Psychology. 2005;10(2):138 doi: 10.1037/1076-8998.10.2.138 15826224 10.1037/1076-8998.10.2.138

[8] Westman M, Etzion D, Danon E. Job insecurity and crossover of burnout in married couples. Journal of Organizational Behavior. 2001;22(5):467–81.

[9] Westman M, Keinan G, Roziner I, Benyamini Y. The crossover of perceived health between spouses. Journal of Occupational Health Psychology. 2008;13(2):168 doi: 10.1037/1076-8998.13.2.168 18393586 10.1037/1076-8998.13.2.168

[10] Song Z, Foo M-D, Uy MA. Mood spillover and crossover among dual-earner couples: a cell phone event sampling study. Journal of Applied Psychology. 2008;93(2):443 doi: 10.1037/0021-9010.93.2.443 18361643 10.1037/0021-9010.93.2.443

[11] Rodríguez-Muñoz A, Sanz-Vergel AI, Demerouti E, Bakker AB. Engaged at work and happy at home: A spillover–Crossover model. Journal of Happiness Studies. 2014;15(2):271–83.

[12] Jee SH, Batty GD, Jang Y, Oh DJ, Oh B-H, Lee SH, et al The Korean Heart Study: rationale, objectives, protocol, and preliminary results for a new prospective cohort study of 430,920 men and women. European journal of preventive cardiology. 2013:2047487313497602. 10.1177/2047487313497602 23864362

[13] Anderson KM, Odell PM, Wilson PW, Kannel WB. Cardiovascular disease risk profiles. American heart journal. 1991;121(1):293–8. 1985385 10.1016/0002-8703(91)90861-b

[14] Jee SH, Jang Y, Oh DJ, Oh B-H, Lee SH, Park S-W, et al A coronary heart disease prediction model: the Korean Heart Study. BMJ open. 2014;4(5):e005025 doi: 10.1136/bmjopen-2014-005025 24848088 10.1136/bmjopen-2014-005025 PMC4039825

[15] Newman DA. Missing data: Five practical guidelines. Organizational Research Methods. 2014;17(4):372–411.

[16] Grzywacz JG, Demerouti E. New frontiers in work and family research. Hove, UK: Psychology Press; 2013.

[17] Westman M. Stress and strain crossover. Human Relations. 2001;54(6):717–51.

[18] Shimazu A, Kubota K, Bakker A, Demerouti E, Shimada K, Kawakami N. Work-to-family Conflict and Family-to-work Conflict among Japanese Dual-earner Couples with Preschool Children: A Spillover-Crossover Perspective. Journal of occupational health. 2013;55(4):234–43. 23748206 10.1539/joh.12-0252-oa

[19] Matthews RA, Del Priore RE, Acitelli LK, Barnes-Farrell JL. Work-to-relationship conflict: crossover effects in dual-earner couples. Journal of Occupational Health Psychology. 2006;11(3):228 doi: 10.1037/1076-8998.11.3.228 16834471 10.1037/1076-8998.11.3.228

[20] Repetti R, Wang S-w, Saxbe D. Bringing It All Back Home How Outside Stressors Shape Families' Everyday Lives. Current Directions in Psychological Science. 2009;18(2):106–11.

[21] Saxbe D, Repetti RL. For better or worse? Coregulation of couples’ cortisol levels and mood states. Journal of personality and social psychology. 2010;98(1):92 doi: 10.1037/a0016959 20053034 10.1037/a0016959

[22] Demerouti E, Bakker AB, Schaufeli WB. Spillover and crossover of exhaustion and life satisfaction among dual-earner parents. Journal of Vocational Behavior. 2005;67(2):266–89.

[23] Westman M, Vinokur AD. Unraveling the relationship of distress levels within couples: Common stressors, empathic reactions, or crossover via social interaction? Human Relations. 1998;51(2):137–56.

[24] Spitze G. Women's employment and family relations: A review. Journal of Marriage and the Family. 1988:595–618.

[25] Raley S, Bianchi SM, Wang W. When Do Fathers Care? Mothers’ Economic Contribution and Fathers’ Involvement in Child Care1. American Journal of Sociology. 2012;117(5):1422–59. 10.1086/663354 PMC4568757 26379287

[26] Dewe P, Hart P, Lu L. A crossnational comparative study of work/family stressors, working hours, and well-being: China and Latin America vs. the Anglo world. Personnel Psychology. 2004;57.

