# Supplementary material for: Crossover effect of spouse weekly working hours on estimated 10-years risk of cardiovascular disease
Source: PLoS One. 2017 Aug 3;12(8):e0182010. doi: 10.1371/journal.pone.0182010 (PMC5542474; doi:10.1371/journal.pone.0182010)
Supplement: S1 Table — (DOCX) [file pone.0182010.s001.docx]

|  | **Spouse's Working Hours** | **N** | **10-year risk** | | |  |  | **≥ 90 percentile of estimated risk of cardiovascular disease** | | | |
| --- | --- | --- | --- | --- | --- | --- | --- | --- | --- | --- | --- |
|  |  |  | **Mean** |  | **SD** |  |  | **N** | **%** | **OR*** | **95% CI** |
| Husband’s 10-year Risk of CVD According Wives' Working Hour Categories | <30 | 1277 | 1.77 |  | 1.74 |  |  | 116 | 9.08 | 1.25 | 0.97-1.55 |
|  | 30-39 | 858 | 1.68 |  | 1.73 |  |  | 74 | 8.62 | 1.41 | 1.11-1.80 |
|  | 40 | 779 | 1.22 |  | 1.19 |  |  | 32 | 4.11 | 1 | Reference |
|  | 40-49 | 925 | 1.62 |  | 1.57 |  |  | 64 | 6.92 | 1.79 | 1.41-2.27 |
|  | 50-59 | 666 | 1.79 |  | 1.54 |  |  | 55 | 8.26 | 1.95 | 1.52-2.50 |
|  | 60-69 | 435 | 2.05 |  | 1.90 |  |  | 53 | 12.18 | 1.92 | 1.46-2.51 |
|  | 70-79 | 346 | 1.83 |  | 1.31 |  |  | 32 | 9.25 | 1.58 | 1.17-1.13 |
|  | ≥80 | 217 | 2.14 |  | 1.73 |  |  | 20 | 9.22 | 2.40 | 1.74-3.31 |
|  | Total | 5503 | 1.76 |  | 1.79 |  |  | 446 | 8.10 | *p for trend<.0001* | |
| Wife’s10-year Risk of CVD According husband’s Working Hour Categories | <30 | 872 | 0.91 |  | 1.07 |  |  | 178 | 20.41 | 5.33 | 3.97-7.16 |
|  | 30-39 | 795 | 0.53 |  | 0.77 |  |  | 74 | 9.31 | 3.71 | 2.71-5.06 |
|  | 40 | 1530 | 0.25 |  | 0.50 |  |  | 28 | 1.83 | 1 | Reference |
|  | 40-49 | 1748 | 0.31 |  | 0.57 |  |  | 69 | 3.95 | 2.00 | 1.47-2.73 |
|  | 50-59 | 1738 | 0.33 |  | 0.60 |  |  | 63 | 3.62 | 2.03 | 1.49-2.77 |
|  | 60-69 | 1163 | 0.33 |  | 0.61 |  |  | 54 | 4.64 | 2.86 | 2.10-3.90 |
|  | 70-79 | 706 | 0.43 |  | 0.95 |  |  | 35 | 4.96 | 1.98 | 1.41-2.77 |
|  | ≥80 | 414 | 0.43 |  | 0.69 |  |  | 26 | 4.93 | 2.37 | 1.65-3.40 |
|  | Total | 8966 | 0.54 |  | 0.90 |  |  | 527 | 5.88 | *p for trend (≥40 hrs)=0.0002* | |

**Supplementary TABLE 1.** 10-year risk of CHD estimated by Jee's appraisal model according to spouse working hour categories including subjects with missing values, which are treated by multiple imputation.

*adjusted for household income level, employment status, own working hours and spouse occupation categories
