# Supplementary material for: Crossover effect of spouse weekly working hours on estimated 10-years risk of cardiovascular disease
Source: PLoS One. 2017 Aug 3;12(8):e0182010. doi: 10.1371/journal.pone.0182010 (PMC5542474; doi:10.1371/journal.pone.0182010)
Supplement: S2 Table — (DOCX) [file pone.0182010.s002.docx]

**Supplementary Table 2.** Cardiovascular risk profiles used in Jee's appraisal model according to spouse working hour categories

| Spouse's Weekly working hours | SBP | |  | DBP | |  | TC | |  | HDL | |  | DM | |  | Smoking | |
| --- | --- | --- | --- | --- | --- | --- | --- | --- | --- | --- | --- | --- | --- | --- | --- | --- | --- |
|  | Mean | SD |  | Mean | SD |  | Mean | SD |  | Mean | SD |  | n | % |  | n | % |
| Male |  |  |  |  |  |  |  |  |  |  |  |  |  |  |  |  |  |
| <30 | 121.25 | 15.93 |  | 79.11 | 10.84 |  | 190.49 | 35.03 |  | 48.62 | 12.23 |  | 119 | 10.85 |  | 473 | 40.74 |
| 30~40 | 121.28 | 15.61 |  | 79.52 | 10.58 |  | 193.17 | 34.56 |  | 48.99 | 12.07 |  | 67 | 8.96 |  | 357 | 44.68 |
| 40 | 119.52 | 15.55 |  | 80.31 | 10.70 |  | 191.53 | 33.93 |  | 47.82 | 10.94 |  | 59 | 8.63 |  | 326 | 45.59 |
| 40~50 | 121.36 | 15.61 |  | 79.43 | 10.66 |  | 192.32 | 34.04 |  | 48.68 | 12.04 |  | 114 | 14.02 |  | 378 | 44.21 |
| 50~60 | 122.82 | 16.55 |  | 80.19 | 10.75 |  | 189.55 | 35.42 |  | 47.87 | 11.70 |  | 71 | 11.93 |  | 259 | 41.64 |
| 60~70 | 122.90 | 16.55 |  | 79.61 | 11.56 |  | 190.28 | 35.22 |  | 48.90 | 12.79 |  | 55 | 14.10 |  | 188 | 46.53 |
| 70~80 | 124.96 | 17.27 |  | 80.36 | 11.27 |  | 190.00 | 36.64 |  | 49.26 | 12.99 |  | 44 | 14.10 |  | 152 | 46.91 |
| ≥80 | 122.85 | 16.24 |  | 79.31 | 10.97 |  | 186.90 | 36.98 |  | 47.68 | 11.62 |  | 39 | 19.80 |  | 87 | 41.83 |
| Total | 121.66 | 15.96 |  | 79.44 | 10.66 |  | 190.26 | 34.96 |  | 48.07 | 11.87 |  | 568 | 11.75 |  | 2220 | 43.63 |
| Female |  |  |  |  |  |  |  |  |  |  |  |  |  |  |  |  |  |
| <30 | 122.08 | 18.41 |  | 75.69 | 9.88 |  | 197.50 | 36.61 |  | 52.41 | 13.26 |  | 84 | 10.87 |  | 26 | 3.13 |
| 30~40 | 116.11 | 18.29 |  | 74.04 | 10.00 |  | 192.56 | 36.47 |  | 53.75 | 12.30 |  | 49 | 6.84 |  | 20 | 2.62 |
| 40 | 110.96 | 15.21 |  | 72.95 | 9.94 |  | 188.62 | 34.22 |  | 55.27 | 12.54 |  | 62 | 4.38 |  | 40 | 2.70 |
| 40~50 | 112.88 | 16.60 |  | 73.41 | 9.98 |  | 187.83 | 35.58 |  | 54.73 | 12.59 |  | 78 | 4.89 |  | 58 | 3.46 |
| 50~60 | 112.74 | 15.70 |  | 73.52 | 9.98 |  | 187.86 | 37.67 |  | 54.19 | 12.72 |  | 73 | 4.55 |  | 58 | 3.45 |
| 60~70 | 113.84 | 16.49 |  | 73.87 | 9.98 |  | 187.47 | 34.19 |  | 53.98 | 12.22 |  | 53 | 4.97 |  | 45 | 4.03 |
| 70~80 | 115.07 | 16.71 |  | 74.42 | 10.09 |  | 190.25 | 37.31 |  | 53.37 | 12.33 |  | 46 | 7.04 |  | 29 | 4.26 |
| ≥80 | 115.33 | 17.24 |  | 74.22 | 10.11 |  | 189.96 | 34.61 |  | 53.03 | 12.74 |  | 23 | 6.22 |  | 19 | 4.82 |
| Total | 115.85 | 17.44 |  | 74.13 | 10.04 |  | 190.71 | 36.31 |  | 53.75 | 12.64 |  | 468 | 5.71 |  | 295 | 3.42 |
